# Molecular Characterization of *Trypanosoma cruzi* from Triatomine Species in São Paulo State, an Area Free of Vector-Borne Chagas Disease

**DOI:** 10.3390/insects16020161

**Published:** 2025-02-04

**Authors:** Eliana Ferreira Monteiro, Rubens Antonio da Silva, Arlei Marcili, Karin Kirchgatter

**Affiliations:** 1 Laboratório de Bioquímica e Biologia Molecular, Instituto Pasteur, São Paulo 01027-000, SP, Brazil; elianafmonteiro@alumni.usp.br; 2 Laboratório de Triatomíneos—Mogi Guaçu, Instituto Pasteur, Mogi Guaçu 13845-206, SP, Brazil; 3 Departamento de Medicina Veterinária Preventiva e Saúde Animal, Universidade de São Paulo, São Paulo 05508-270, SP, Brazil; 4 Programa de Medicina e Bem-Estar Animal e Saúde Única, Universidade Santo Amaro, São Paulo 04829-300, SP, Brazil

**Keywords:** Chagas disease, trypanosomatid, *Trypanosoma cruzi*, *Rhodnius neglectus*, *Triatoma sordida*, São Paulo state

## Abstract

Chagas disease (ChD) is a neglected tropical disease caused by *Trypanosoma cruzi*, endemic in 21 countries across the Americas, with increasing cases globally. In São Paulo, Brazil, vector control has focused on *Triatoma infestans*, but secondary triatomine species continue to pose transmission risks. This study aimed to investigate the prevalence of *T. cruzi* in triatomine feces and characterize its genetic diversity using molecular techniques. Fecal samples were collected from 570 triatomines across 25 municipalities in São Paulo, followed by DNA extraction and PCR amplification targeting the mitochondrial cytochrome b gene and the V7V8 region of the 18S rRNA gene. The results revealed a low overall infection rate (3.2%). However, excluding the triatomines collected in palm trees, all of which were negative, we found mainly *Panstrongylus megistus* in residences and peridomiciles, showing the highest infection rate (65%) for *T. cruzi*, followed by *Triatoma sordida* and *Rhodnius neglectus*. Phylogenetic analysis confirmed that DTU TcI was the most prevalent genotype, consistent with previous findings in the region. This study highlights the importance of continued vector surveillance, as these secondary species are capable of maintaining *T. cruzi* transmission in both urban and rural environments, underscoring the ongoing risk of ChD resurgence in São Paulo.

## Simple Summary

This study focused on understanding how common the parasite *Trypanosoma cruzi* (which causes Chagas disease) is in certain insects called triatomines, by analyzing their feces. Samples were collected from 570 triatomines in 25 cities in São Paulo and tested for the parasite’s DNA. A low overall infection rate was found (3.2%); however, excluding the insects collected in palm trees, where no positives were found, the infection rate was very high among those collected in residences and peridomiciles (45%), the majority of which were *Panstrongylus megistus*, and 65% of them were positive for *T. cruzi*. These findings are important because although the main vectors of Chagas disease have been controlled, other insect species are still capable of spreading the disease, especially in both city and rural areas. This highlights the need for ongoing monitoring of these insects to prevent a resurgence of Chagas disease in São Paulo, protecting public health.

## 1. Introduction

Chagas disease (ChD) is a parasitic disease caused by *Trypanosoma cruzi* [1] and is listed among the 20 neglected tropical diseases [2]. This disease is endemic in 21 countries in the Americas, and it is estimated that approximately 70 million people live in places at risk for infection, with 6 million people affected worldwide [3]. In Latin America, there is an annual incidence of 30 thousand new cases, causing, on average, 14 thousand deaths/year [4]. The Pan American Health Organization has recognized the eradication of household transmission of *T. cruzi* in Uruguay (1997), Chile (1999), Brazil (2006), and Paraguay (2013); however, transmission still occurs in other Latin American countries [3]. In São Paulo State, vector-borne ChD transmission was eliminated in the 1970s [5]. However, in recent years, human cases have been detected in countries such as Japan and Spain [6], the United States [7], and even in the city of São Paulo [8], a reflection of commercial exchanges and the flow of people between different continents.

*Trypanosoma cruzi* is a flagellated protozoan belonging to the order *Kinetoplastida*, family Trypanosomatidae, which can be transmitted mainly to humans by triatomines [9]. In the state of São Paulo, the history of vector-borne ChD was a model for Brazil and countries in Latin America [5]. With the success in controlling and eliminating *Triatoma infestans*, one of the main vector species, entomological surveillance and control activities were directed at wild species that occasionally invade residences and can form colonies in their surroundings [10]. As São Paulo is officially recognized as a “free area” for vector transmission of *T. cruzi* by its primary vector species, secondary or wild species of triatomines can still carry and transmit *T. cruzi*, which represents a risk of resurgence or isolated cases of infection [11]. Thus, the detection and molecular characterization of *T. cruzi* in these species is relevant for understanding the dynamics of ChD in regions where vector transmission has been under control or eradicated.

Like most species of protozoa, the parasite *T. cruzi* circulates in two types of alternating hosts to complete its life cycle, with the invertebrate host being hematophagous insects that act as a vector. *Trypanosoma cruzi* is distributed in several genera of the Triatominae family and dozens of triatomine species. Its vertebrate hosts are numerous, with more than a hundred species of mammals [12,13].

Of the 14 triatomine species already found in the state of São Paulo, the most frequently found are *Triatoma sordida*, *Panstrongylus megistus*, *Rhodnius neglectus*, and *Triatoma tibiamaculata* (=*Panstrongylus tibiamaculatus*) [10,14]. However, *Panstrongylus megistus* is one of the triatomine species of greatest epidemiological interest due to its proximity to human habitations, as it is capable of colonizing indoors and outdoors, and due to the high rates of infection by *T. cruzi* [15].

Currently, a classification of six Discrete Typing Units (DTUs) (TcI-TcVI) is recognized, with DTU TcI corresponding to *T. cruzi* group I in the new nomenclature, and *T. cruzi* group II being subdivided into four DTUs (TcII-TcVI) [16]. The seventh DTU of *T. cruzi* associated with a new genotype found in some Brazilian bat species was characterized in 2009, known as DTU TcBat [17], and subsequently reported in bats from other South American countries [18,19].

DTU TcI is one of the most abundant and widely dispersed DTUs. Throughout the parasite’s range, it is frequently isolated in all taxa of wild mammals, covering a wide diversity of biomes and habitats, and may be associated with both wild and domestic cycles [20]. DTU TcII is found predominantly in the southern and central regions of South America, having been isolated mainly from domestic transmission cycles; however, most reported isolations were made from primates and sporadically from other species of wild mammals in remaining fragments of the South Atlantic Forest, Brazil [20].

Molecular tools such as PCR (polymerase chain reaction) are employed to detect and identify *T. cruzi* DNA in triatomine insects. These techniques provide greater sensitivity than traditional microscopy and can identify infections at low parasitemia levels. In this study, the objective was to investigate, in the state of São Paulo, Brazil, the rate of natural infection by *T. cruzi* in triatomine feces of different species, through molecular diagnosis using the variable V7–V8 region of ssrDNA. Secondarily, we aimed to infer the genetic relatedness between isolates from these hosts by comparing sequences to those found in the main *T. cruzi* vector. With this analysis, we sought to answer some important questions, such as which triatomine species have a higher prevalence of infection and which live near humans and thus offer a great risk of infection, given the importance of epidemiological surveillance in controlling ChD transmission.

## 2. Materials and Methods

### 2.1. Samples

**Figure 1:**
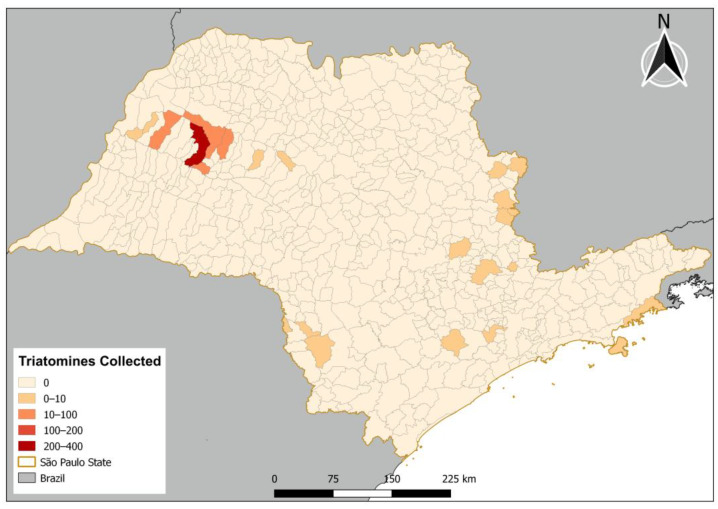
Distribution of triatomines collected in the state of São Paulo.

Triatomine samples were collected in 25 municipalities in the state of São Paulo during the period from June 2021 to September 2022 (Figure 1).

These collections were performed in urban and rural environments. In the urban environment, the collection was carried out on palm trees, which have been found to be infested by triatomines of the species *Rhodnius neglectus* in municipalities in the northwest region of the state of São Paulo, following an established protocol carried out every year [21]. This collection was carried out in four municipalities (Araçatuba, Birigui, Guararapes, and Piacatu), with a total of 521 triatomines. With the help of a Munck truck, agents were taken to the tops of the palm trees to handle and remove organic material produced by these trees, as well as to check for the presence of triatomines in bird nests, which have been identified as propagators of these insects in this environment [21]. Another collection was carried out in the rural environment, where this research followed state regulations, responding to resident reports of possible triatomine findings. An active search was carried out on the properties from which the notifications originated, with research directed at locations with possible food sources for triatomines, such as chicken coops [10]. This collection was carried out in all the other municipalities analyzed in this study (21 counties), with a total of 49 triatomines. Thus, in total, 570 triatomines from five different species were collected: *P. megistus*, *P. diasi*, *Rhodnius neglectus*, *Triatoma sordida*, and *Triatoma tibiamaculata* (=*Panstrongylus tibiamaculatus*) (Table 1).

The feces were removed from the triatomines and impregnated on 3M Whatman^®^ filter paper. The fecal content of the triatomine was obtained through delicate compression on the insect’s abdomen, and the material obtained was mixed with a drop of saline solution and absorbed onto 2.0 cm × 0.5 cm strips of 3M Whatman^®^ filter paper. The filter papers were maintained at room temperature for drying and further stored in individual 1.5 mL tubes.

### 2.2. Genomic DNA Extraction

The extraction of genomic DNA from the 570 triatomine fecal samples preserved on filter paper was carried out using the DNeasy Blood & Tissue Kit (Qiagen, Germantown, MD, USA). A pre-lysis was performed using the ASL Buffer manufactured by Qiagen (Qiagen, Germantown, MD, USA), adding 220 µL of ASL buffer to each microtube containing the filter paper with feces. Then, the samples were heated to 70 °C and remained under stirring at 400 rpm in a Vortemp™ 1550 Shaking Incubator (Labnet, Edison, NJ, USA) for 15 min. After natural cooling to approximately 56 °C, 20 µL of Proteinase K and 200 µL of AL buffer were added. The samples were heated again to 56 °C and remained under stirring at 400 rpm in a Vortemp™ 1550 Shaking Incubator (Labnet, Edison, NJ, USA) for 2 h. The samples were incubated at 37 °C and remained under agitation at 400 rpm in a Vortemp™ 1550 Shaking Incubator (Labnet, Edison, NJ, USA) overnight. An aliquot of 200 μL of absolute ethanol was added to each sample; then, the DNeasy Blood & Tissue Kit (Qiagen, Germantown, MD, USA) was used according to the manufacturer’s instructions.

### 2.3. Molecular Analysis

Samples extracted from triatomines were analyzed using Polymerase Chain Reaction (PCR) targeting the mitochondrial gene cytochrome b (*cytb*) of *T. cruzi* using the primers p18 (GAC AGG ATT GAG AAG CGA GAG AG) and p20 (CAA ACC TAT CAC AAA AAG CAT CTG) [22] in a final volume of 25 μL containing 0.5 units of Taq DNA Polymerase Platinum (Invitrogen), 0.1 nM of each dNTP, 0.2 mM of each primer, 1.5 mM MgCl_2_, 1X buffer, and 5 μL of gDNA. Cycling was performed according to the established protocol for the amplification of a fragment of approximately 722 bp: 94 °C for 5 min; 35 amplification cycles: 94 °C for 1 min; 50 °C for 30 s; 72 °C for 90 s; and a final extension of 72 °C for 5 min [23].

The second target evaluated in this work was the V7V8 SSU hypervariable region of the gene that encodes the smaller subunit of ribosomal RNA, also known as 18S, which is a region widely used for phylogenetic analysis within the Trypanosomatidae group [24]. For this analysis, a semi-nested-PCR protocol was adapted, where the 609F (5′ CAC CCG CGG TAA TTC CAG C 3′) and 706R (5′ CTG AGA CTG TAA CCT CAA 3′) primers were used in the first reaction, under the following conditions: 94 °C for 5 min; 34 cycles: 94 °C for 1 min; 48 °C for 2 min; 72 °C for 2 min; and a final extension of 72 °C for 10 min. This produced a fragment of approximately 940 bp. For the second reaction, the triatomine amplicons were diluted 1:500 followed by the semi-nested-PCR reaction with the primers SSU_2F (5′ CCA AAG CAG TCA TCC GAC TT 3′) and 706R (5′ CTG AGA CTG TAA CCT CAA 3′), producing an internal fragment of 688 bp. 

The amplified products were purified with ExoSAP-IT™ (Applied Biosystems, Carlsbad, CA, USA), according to the manufacturer’s instructions. All the purified fragments were subjected to the sequencing reaction using the BigDye Terminator v3.0 Cycle Sequencing Kit (Applied Biosystems, Carlsbad, CA, USA), according to the manufacturer’s instructions. The runs were performed on the ABI PRISM^®^ 3130XL Genetic Analyzer Sequencer (Applied Biosystems, Carlsbad, CA, USA) from the Chemistry Institute of the University of São Paulo.

### 2.4. Phylogenetic Analysis

The phylogenetic relationships among the reported parasites were deduced by analyzing partial 18S ribosomal RNA gene sequences (~700 bp). GenBank accession numbers for the sequences used in the phylogenetic reconstruction are provided in the phylogenetic trees. Bayesian inference, implemented in MrBayes v3.2.0, was employed for the phylogenetic reconstructions [25]. The alignment was obtained using the MUSCLE algorithm implemented in MEGAX version 10.0.5 [26]. Bayesian inference was conducted through two Markov Chain Monte Carlo searches comprising 3 million generations, with a sampling of 1 out of every 300 trees. Following the removal of a burn-in of 25%, the remaining trees were utilized to generate the 50% majority-rule consensus tree. The resultant phylogenetic tree was then visualized using FigTree version 1.4.0 [27].

## 3. Results

A total of 570 triatomine samples from the state of São Paulo were analyzed for the detection of trypanosomatids, with 91.4% collected in palm trees, all identified as *R. neglectus*. In the captures carried out by the Pasteur Institute field team, by an active search after resident reports, 49 triatomines were found (8.6%) in 21 municipalities of the state of São Paulo. *P. megistus* was the most recurrent, corresponding to 53% of the specimens captured, and *T. sordida* occupied the second position with 40% (Table 1).

Three species of triatomines, previously identified by morphology, were positive: *Panstrongylus megistus*, *Triatoma sordida*, and *Rhodnius neglectus* (Table 1). *Panstrongylus megistus* was the species that presented the highest positivity rate, reaching 65% detection of *T. cruzi* among the 26 specimens collected. All the *T. cruzi*-positive samples were found by an active search after resident reports. These captures, carried out by the Pasteur Institute field team, registered 44% positivity. All the samples obtained from the triatomines collected in palm trees (521 samples from *Rhodnius neglectus*) were negative for trypanosomatids (Table 1).

The assay for amplification of the mitochondrial gene cytochrome b (*cytb*) detected the presence of *T. cruzi* in 3.2% of the samples analyzed (Table 1). DNA sequencing was carried out to confirm positivity for *T. cruzi* in the triatomine samples. DNA products obtained from the amplification reaction for the *cytb* gene were sequenced with an average length of 630 bp.

Amplification of the V7V8 region of the 18S gene showed a positivity of 3.9% (22 of 570) in the samples analyzed. It is possible to observe in Table 1 that the V7V8 protocol detected *T. cruzi* in the same 18 samples positive for *cytb* and, additionally, in four other samples, two of which were identified as *T. cruzi*, and the other two were yet unidentified trypanosomatids.

Regarding the molecular analysis, a total of 17 PCR-positive samples for the 18S gene (14 from *T. cruzi* obtained from *P. megistus*, 1 from *R. neglectus*, and 2 from *T. sordida*) were sequenced successfully and used for further analysis. These sequences presented good criteria for use in the construction of the phylogenetic tree. The other samples did not show clear electropherograms (without double peaks) and were removed. Eight sequences (five from *T. cruzi* in *P. megistus*, one from *T. cruzi* in *R. neglectus*, and two from *T. cruzi* in *T. sordida*) were identical and showed high identity (99%, 689/690 bp), with a sequence of *T. cruzi* obtained from *P. megistus* collected in Mogi Guaçu (KF788250). Five sequences (all from *T. cruzi* in *P. megistus*) presented high identity with *T. cruzi* strain Yuyu (AF245380, 99%, 695/697 bp).

**Figure 2:**
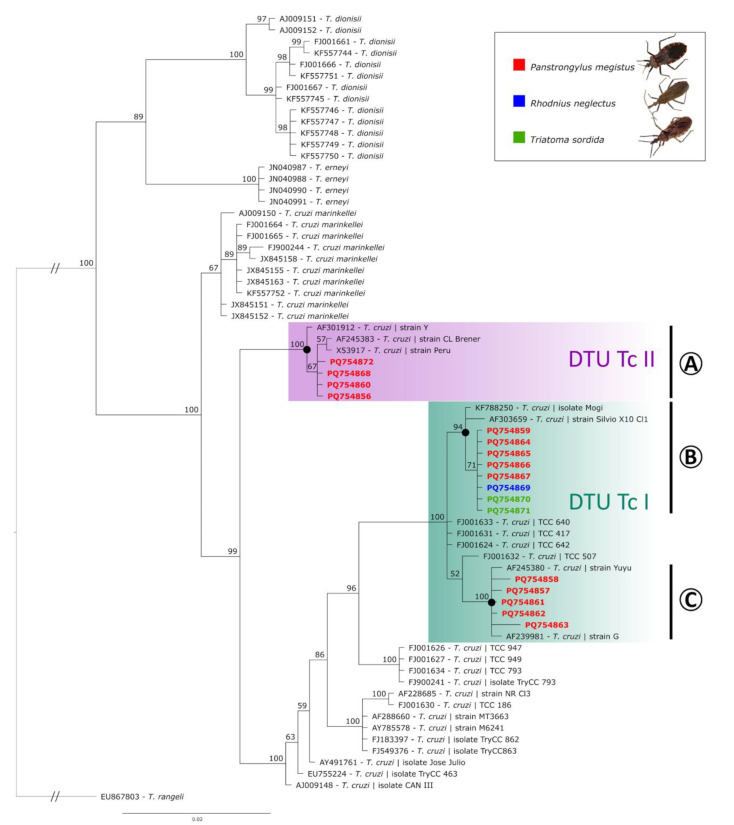
Bayesian phylogeny based on the partial 18S ribosomal RNA sequences of *Trypanosoma* species (alignment of 758 bp). *Trypanosoma rangeli* was used as an outgroup. Support values on nodes (in percentage) indicate posterior probabilities (PPs). Sequences obtained in this study are indicated according to the hosts: *Panstrongylus megistus* (red), *Rhodnius neglectus* (blue), and *Triatoma sordida* (green). DTU TcII (**A**) and DTU TcI (**B**,**C**) clades are shown.

The final alignment used for phylogenetic analysis combined the 17 *T. cruzi* sequences detected in this study with 52 previously recorded sequences from the GenBank database, which were either closely matched to the sequences obtained here or represented related *Trypanosoma* species. The Bayesian analysis (average standard deviation of split frequencies: 0.004199) resulted in a tree containing distinct clades according to the different *Trypanosoma* species (Figure 2). First, *Trypanosoma dionisii* sequences were grouped in a clade with 100% of posterior probability (PP). *Trypanosoma erneyi* sequences were also positioned in a group with high PP (81%). The sequences obtained from *T. cruzi marinkellei* were grouped in a sister clade of *T. cruzi*, with 100% PP (Figure 2). All the *T. cruzi* sequences obtained from this study were correctly grouped in the *T. cruzi* clade, which had a separate clade (100% PP), composed of one sequence of *T. cruzi* strain Y from humans (AF301912), one sequence of *T. cruzi* strain Peru (from D. McMahon-Pratt, Yale University MedicalSchool, X53917), one sequence of *T. cruzi* from *Triatoma infestans* (CL Brener strain, AF245383), and four sequences obtained in this study of *T. cruzi* from *P. megistus* (from Ilhabela and Campinas) (clade A, DTU TcII, Figure 2). Most of the sequences obtained in this study (76.5%) were branched along with other Brazilian sequences, with strongly supported clades solely composed of sequences reported in Brazil, split into two subclades composed of (i) KF788250 (*T. cruzi* isolate from *P. megistus* collected in Mogi Guaçu), AF303659 (*T. cruzi* strain Silvio X10), and nine sequences from this study (*T. cruzi* obtained in *P. megistus*, *R. neglectus*, and *T. sordida* from Taboão da Serra, Sto. Antonio Jardim, Barão de Antonina, Piedade, Itaberá, Campinas, and Mirandópolis) (clade B, DTU TcI, Figure 2) and (ii) four *T. cruzi* sequences from *P. megistus* (from Limeira, São José do Rio Pardo and Cotia) positioned close to sequence AF239981 (*T. cruzi* strain G) (clade C, DTU TcI, Figure 2).

**Figure 3:**
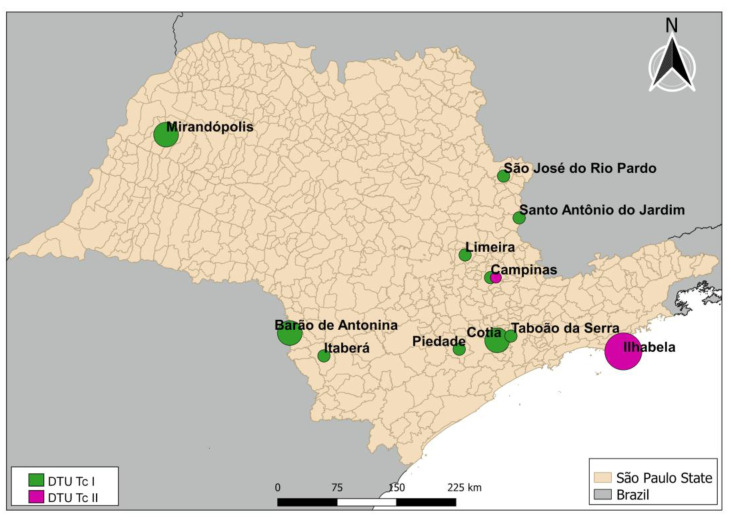
Distribution of positive samples detected in the state of São Paulo. Circle sizes represent the number of triatomines positive for *Trypanosoma cruzi*. The smallest circle represents one triatomine, and the largest circle represents three specimens.

The 17 samples that were positive for *T. cruzi* and characterized according to DTU classification, were found in a sparse distribution among 11 municipalities in the state, as shown in Figure 3.

## 4. Discussion

The circulation of *Trypanosoma cruzi* in the state of São Paulo has been decreasing, despite new areas of occurrence [11]. In this study, we demonstrated the circulation of trypanosomatid species among triatomines. Changes in the distribution pattern of species, with occupation in urban environments, have been reflected in the density of vectors, with *R. neglectus* being one of the most collected species, in captures carried out by municipalities [28].

*Rhodnius neglectus* is the most widespread species in Brazil, occurring in 13 Brazilian states (Acre, Bahia, Goiás, Maranhão, Mato Grosso, Mato Grosso do Sul, Minas Gerais, Paraíba, Pernambuco, Paraná, Piauí, São Paulo, Tocantins), and it is mainly associated with palm trees throughout the Cerrado and the southern margin of the Amazon [29]. The explanation for this large distribution may be the facilitation of passive dispersal by birds, through the mechanism of adherence of eggs and nymphs to feathers [29]. In our study, none of the 521 insects of this species collected in palm trees were positive, while only one individual collected in a forest area was found positive for *T. cruzi*. In the wild, *R. neglectus* primarily feeds on birds, with rodents being a less common blood source, and opossums are rarely bitten [28], which can justify the fact that this species of triatomine plays a role of secondary importance in the transmission of *T. cruzi* [30].

After the elimination of the transmission of ChD by *T. infestans*, which occurred between the late 1960s and early 1970s in the state of São Paulo, *P. megistus* is considered the main vector of *T. cruzi* in Brazil [31]. It has a wide geographic distribution, high rates of natural infection, and easily colonizes artificial ecotopes. Due to human action in the environment and the reduction in its food sources, this vector is present in homes [32]. In this study, *P. megistus* presented the highest rate of infectivity by *T. cruzi* in both molecular methods used to detect the parasite, corroborating data from the literature. These high infection rates found for the species can be explained by the fact that these triatomines feed mainly on the blood of rodents and opossums [32].

Another species in which *T. cruzi* could be identified and characterized was *T. sordida*. This species is widely distributed in Brazil and is usually the most commonly collected in the state of São Paulo, but it has the lowest rates of natural infection. Found predominantly in the peridomicile of residences, in chicken coops, or bird nests in the eaves of properties, its association with birds would explain the lower infection rate found [11].

The phylogenetic analysis of this study showed a clear clustering of sequences by DTUs with 100% support, but also evidence of sub-DTU diversity. Although the samples we sequenced were positioned in some subclades with lower support, this sub-structuring has been shown in many studies and is associated, in part, with geographic structuring [33].

Despite the limitations of this study, including its dependence on successful collections or the sending of insects by the population for identification or examination, we were able to demonstrate that DTU TcI is the most common lineage that infects triatomine species in São Paulo State (76%), with 24% belonging to DTU TcII. In this sense, knowing the diversity of *T. cruzi* lineages becomes relevant for public health since genetic subpopulations can influence the clinical evolution of ChD [34]. DTU TcI is the most common in Central America and northern South America, often associated with less severe forms of the disease, while DTU TcII is associated with severe cardiac manifestations in countries of the Southern Cone [35]. Another relevant factor concerns the response to antiparasitic treatment, which is distinctly dependent on the lineage [36].

In addition, knowledge of the lineage allows us to verify the spread of the parasite in different species, as well as identify transmission cycles (wild or domestic). This helps to design more effective control strategies since DTU TcI is more related to wild cycles, while DTU TcII is more associated with domestic cycles [37].

This is the first study characterizing an important number of *T. cruzi* sequences obtained from three triatomine species in São Paulo State by comparing molecular and biogeographical data, including the first sequences of *T. cruzi* described on *Rhodnius neglectus* and *Triatoma sordida* in the state.

## 5. Conclusions

We showed a higher *T. cruzi* infection rate for *P. megistus* but also positive samples on *Rhodnius neglectus* and *Triatoma sordida*, which were collected in different municipalities and distributed mainly in the southeast region of the state of São Paulo. Some *T. cruzi* sequences obtained from *P. megistus* collected in Campinas and Ilhabela were grouped together with a sequence of *T. cruzi* from *Triatoma infestans*, the primary vector species of ChD transmission.

The presence of vectors naturally infected by *T. cruzi* is directly related to the risk of transmission of the etiological agent and the occurrence of new cases of ChD. This work indicates the presence of infected vectors in urban and rural environments in the state of São Paulo, reaffirming the need and importance of vector surveillance work, through actions that can prevent the transmission of ChD.

The results of this study are crucial for improving the understanding of *T. cruzi* infection dynamics in triatomine species and their role in ChD transmission in São Paulo State. By identifying which triatomine species exhibit higher infection rates and assessing their proximity to human populations, this study provides valuable insights for developing targeted surveillance systems and intervention strategies. This research is especially important for addressing the potential zoonotic reservoir in densely populated areas, helping to prevent further human infections and improve public health outcomes in the region.

## Figures and Tables

**Table 1:** Detection of trypanosomatids in different triatomine species using two molecular protocols, by collection method.

	Analyzed		Positives ^#^				
			*cytb* (%)		V7V8 (%)		
Triatomine Species	Palm Trees	Other Sites *	Palm Trees	Other Sites *	Palm Trees	Other Sites *	Sequence (#GenBank)
*Panstrongylus diasi*	0	1	0	0	0	0	-
*P. megistus*	0	26	0	16 (61.5)	0	17 (65.4)	*Trypanosoma cruzi* (PQ754856-PQ754868, PQ754872)
*Rhodnius neglectus*	521	1	0	1 (100)	0	1 (100)	*T. cruzi* (PQ754869)
*Triatoma sordida*	0	20	0	1 (5)	0	4 (20)	*T. cruzi* (PQ754870 and PQ754871) + two Trypanosomatidae sp. sequences
*T. tibiamaculata* *(=P. tibiamaculatus)*	0	1	0	0	0	0	-
Total	521	49	0	18 (36.7)	0	22 (44.9)	

* Sites of active search after resident reports of possible triatomines finding. **^#^** Positivity percentages are given in relation to the total analyzed by the collection method.

## Data Availability

The data presented in this study are available in the GenBank database (https://www.ncbi.nlm.nih.gov/genbank/) (accessed on 1 October 2024).
